# An evaluation of global Chikungunya clinical management guidelines: A systematic review

**DOI:** 10.1016/j.eclinm.2022.101672

**Published:** 2022-09-28

**Authors:** Eika Webb, Melina Michelen, Ishmeala Rigby, Andrew Dagens, Dania Dahmash, Vincent Cheng, Reena Joseph, Samuel Lipworth, Eli Harriss, Erhui Cai, Robert Nartowski, Pande Putu Januraga, Keerti Gedela, Evi Sukmaningrum, Muge Cevik, Helen Groves, Peter Hart, Tom Fletcher, Lucille Blumberg, Peter W. Horby, Shevin T. Jacob, Louise Sigfrid

**Affiliations:** a Liverpool School of Tropical Medicine, Pembroke Pl, Liverpool, UK; b International Severe Acute Respiratory and emerging Infection Consortium, Centre for Tropical Medicine, University of Oxford, Oxford, UK; c Imperial College London, London, UK; d Bristol Medical School, University of Bristol, Bristol, UK; e Oxford University Hospitals NHS Foundation Trust, Oxford, UK; f Nuffield Department of Medicine, University of Oxford, Oxford, UK; g Bodleian Health Care Libraries, University of Oxford, Oxford, UK; h Wellcome Trust, Euston Rd, London, UK; i National Institute for Communicable Diseases, Johannesburg, South Africa; j Atma Jaya Catholic University of Indonesia, Jakarta, Indonesia; k HIV AIDS Research Centre-HPSI, AJCU, Jakarta, Indonesia; l Udayana University, Bali, Indonesia; m Department of Global Health and Infection Research, School of Medicine, University of St Andrews, Fife, Scotland, UK

**Keywords:** Clinical management guidelines, AGREE II, Supportive care, Chikungunya, Emerging infections

## Abstract

**Background:**

Chikungunya virus (CHIKV) has expanded its geographical reach in recent decades and is an emerging global health threat. CHIKV can cause significant morbidity and lead to chronic, debilitating arthritis/arthralgia in up to 40% of infected individuals. Prevention, early identification, and clinical management are key for improving outcomes. The aim of this review is to evaluate the quality, availability, inclusivity, and scope of evidence-based clinical management guidelines (CMG) for CHIKV globally.

**Methods:**

We conducted a systematic review. Six databases were searched from Jan 1, 1989, to 14 Oct 2021 and grey literature until Sept 16, 2021, for CHIKV guidelines providing supportive care and treatment recommendations. Quality was assessed using the appraisal of Guidelines for Research and Evaluation tool. Findings are presented in a narrative synthesis. PROSPERO registration: CRD42020167361.

**Findings:**

28 CMGs were included; 54% (15/28) were produced more than 5 years ago, and most were of low-quality (median score 2 out of 7 (range 1–7)). There were variations in the CMGs’ guidance on the management of different at-risk populations, long-term sequelae, and the prevention of disease transmission. While 54% (15/28) of CMGs recommended hospitalisation for severe cases, only 39% (11/28) provided guidance for severe disease management. Further, 46% (13/28) advocated for steroids in the chronic phase, but 18% (5/28) advised against its use.

**Interpretation:**

There was a lack of high-quality CMGs that provided supportive care and treatment guidance, which may impact patient care and outcomes. It is essential that existing guidelines are updated and adapted to provide detailed evidence-based treatment guidelines for different at-risk populations. This study also highlights a need for more research into the management of the acute and chronic phases of CHIKV infection to inform evidence-based care.

**Funding:**

The UK Foreign, Commonwealth and Development Office, Wellcome Trust [215091/Z/18/Z] and the Bill & Melinda Gates Foundation [OPP1209135].

**Research in context**

**Evidence before this study**

Chikungunya virus (CHIKV) is an emerging tropical, mosquito-borne virus, identified in more than 60 countries globally causing regular epidemics predominantly impacting vulnerable populations in lower-resourced settings. To assess availability of clinical management guidelines, their scope, inclusivity, and quality we searched Ovid in Medline, Embase, Global Health, Scopus, Web of Science Core Collection and WHO Global Index Medicus, complemented by a grey literature search and a targeted search of national public health databases in seven languages. The searches were completed on Oct 14, 2021 using a list of terms relating to clinical management guidelines for Chikungunya. We identified 28 CMGs, most of low quality and with limited and at times contradictory treatment advice.

**Added value of this study**

Our data highlights a global scarcity of CMGs for CHIKV providing guidance on optimal care and treatment for different at-risk populations and settings. There was limited guidance available on care for severe cases, and available guidance was heterogenous and discordant (e.g., on use of analgesia, corticosteroids). Moreover, there was limited guidance on referral criteria and level of monitoring of pregnant women and infants at higher risk of severe disease.

**Implications of all the available evidence``**

Our data highlights an urgent need for research into effective treatment strategies to reduce morbidity and prevent risk of long-term sequelae, and for new evidence to be incorporated into clinical management guidelines, for different at-risk populations. We propose development of a harmonised ‘living’ clinical management guideline framework for infectious diseases, to improve standardisation of recommendations, inclusivity, and quality of CMGs, to improve access to evidence based recommendations to improve long term outcomes.

Alt-text: Unlabelled box

## Introduction

Chikungunya is a disease caused by the Chikungunya virus (CHIKV); an arthropod-borne virus transmitted to humans primarily by *Aedes* mosquitoes. Since its description in 1952, CHIKV has caused ten million of human infections.^1^^,^^2^ An outbreak in 2004 affected more than 100 countries with over 10 million cases.^2^ This was followed by another large outbreak across Latin America (2013).^2^ Multiple factors contributed to these outbreaks including limited mosquito control in densely populated urban areas, climate change, and lack of vaccines and effective treatments.^3^ It is estimated that 1.3 billion people live in areas at risk of CHIKV infection,^4^^,^^5^ including Europe.^4^^,^^6^^,^^7^ The recent expansion in geographical range, and localised travel-imported outbreaks have increased CHIKV's recognition as an emerging global health threat.

Chikungunya has a wide spectrum of clinical presentation, classified into three phases (acute, sub-acute and chronic).^8^^,^^9^ Acute CHIKV generally manifests as a febrile illness with predominantly polyarthralgia, rash, and headache. This can be followed by a subacute phase for up to three months**.**^8^^,^^9^ Although the acute infection rarely is life-threatening, it can result in severe illness and mortality in neonates, older adults (over 65 years) and people with comorbidities.^2^^,^^9^, ^10^, ^11^, ^12^ Severe and atypical manifestations include failure of at least one major organ or system and includes neurological, cardiovascular, renal, dermatological and respiratory manifestations,^13^ with life threatening complications such as myocarditis^12^ and encephalitis/encephalopathy^14^ being reported. Pregnant women are at particular risk of severe complications.^15^ The chronic manifestations of CHIKV can affect an estimated 40% of individuals and can include debilitating symptoms of chronic arthralgia, arthritis/arthralgia, fatigue which may lead to disability and diminished quality of life,^16^ with severe impact on an individual's ability to work and can last up to 6 years.^8^^,^^9^^,^^16^, ^17^, ^18^, ^19^, ^20^, ^21^ CHIKV infection is a serious global public health problem, predominantly affecting populations and health systems in lower resourced settings, and with risk of importation into new, naïve regions. Yet, to date, there is no specific treatment approved for CHIKV. Although vaccines have been developed and tested in humans, none are yet available.^22^^,^^23^ Thus, supportive care is essential for improving patient outcomes and reduce the long-term chronic burden.^24^ The aim of this systematic review is to explore the quality, availability, inclusivity, and scope of evidence-based CHIKV clinical management guidelines (CMGs) for different populations globally.

## Methods

### Study design

We defined CMGs as per the World Health Organisation (WHO) definition of a guideline information that  contains recommendations to guide practice, providing recommendations as statements designed to help end-users make informed decisions on whether, when and how to undertake specific actions such as clinical interventions, with the aim of achieving the best possible individual health outcomes.^25^ We conducted a systematic review of Chikungunya CMGs using Cochrane systematic review methodologies,^26^ structured according to the Preferred Reporting Items for Systematic Reviews and Meta-Analyses (PRISMA) guidelines (supplementary file S1 and S2).^27^ The protocol is registered with the International Prospective Register of Systematic Reviews (PROSPERO) (CRD42020167361).^28^

### Search strategy

We conducted a systematic database search through Ovid Medline, Ovid Embase, Ovid Global Health, Scopus, Web of Science Core Collection and WHO Global Index Medicus from inception to 14^th^ October 2021. Search strategies applied the Canadian Agency for Drugs and Technologies in Health (CADTH) database guidelines search filter (supplementary file S1).^29^ Previous reviews have observed that CMGs are not always available indexed in databases. Therefore, we also performed a grey literature search up to 16^th^ September 2021. We searched Google and Google Scholar using predefined keywords in Arabic, English, French, German, Mandarin, Russian, and Spanish. We also contacted expert members of the International Severe Acute Respiratory and emerging Infection Consortium (ISARIC) network requesting CMGs.^30^ A full search strategy is presented in the supplementary file.

### Eligibility criteria

We included Chikungunya CMGs that provided treatment and/or supportive care recommendations. There were no language limitations. We excluded CMGs providing solely recommendations on diagnostics, animal health, or public health. The most recent versions of CMGs were included.

### Screening and data extraction

Search results were screened by title and abstract, followed by full text by two reviewers using the Rayyan systematic review software.^31^ Data was extracted by one reviewer using a standardised Excel form and validated by a second reviewer (Supplementary file S2). We extracted data on bibliography, populations covered, supportive care treatment recommendations and preventive measures to reduce community transmission. Disagreements were resolved via consensus or by a third reviewer. Non-English language CMGs were translated using Google translate and screened, and data extracted by reviewers with good to excellent knowledge of the language.

### Quality appraisal

Quality was assessed independently by two reviewers using the Appraisal of Guidelines for Research and Evaluation II (AGREE II) Instrument.^32^ This tool provides an objective framework for assessing the guideline development process and quality. It is s a 23-item tool that spans six domains comprising different aspects: 1) scope and purpose; 2) stakeholder involvement; 3) rigour of development; 4) clarity of presentation; 5) applicability and 6) editorial independence. Each domain has several sub-criteria which assess whether the criteria are met using a seven-point Likert scale, from 1 (strongly disagree) to 7 (strongly agree). A score of 100% is achieved if the CMG scores 7 for all domain items; 0% if each reviewer scored 1 for all items. When there was limited information about the methodology presented, efforts were made to search for additional information via associated webpages. The final score for each domain was calculated as per the AGREE II domain formula.^32^

CMGs were considered of high quality if they scored more than 60% in domain three (rigour of development; as this is considered a high-quality indicator) and two other non-specified domains moderate quality if they scored more than 60% in any three or more domains but not in domain three; low quality if they did not meet these criteria. Additionally, each CMG was given an overall guideline assessment score based on the domain scores, ranging from one to seven (a score ≥6 = high quality; 4–5 = medium quality; ≤ 3 = low quality) together with a recommendation for use with or without further modifications.^32^ CMGs with a total overall quality score of 1 were not recommended for use, total overall scores of 2–5 were recommended for use with modifications and 6–7 recommended for use without modifications.^32^

$$\text{AGREE}\;\text{II}\;\text{domain}\;\text{calculation}\;\text{formula}:\;\frac{obtained\;score-minimum\;possible\;score}{Maximum\;possible\;score-minimum\;possible\;score}$$

### Data analysis

The availability of CMGs was assessed by whether open-sourced CMGs could be identified and stratified by origin/producer: (1) international and regional organisations (e.g., WHO; Pan-American Health organisation (PAHO)); (2) national organisations (e.g., Ministries of Health) and (3) clinical reference websites (e.g., Medscape, UptoDate). We assessed inclusivity based on inclusion of recommendations for different at-risk groups, including infants/children, pregnant women, older people, and people living with HIV or those with comorbidities. The ggplot2 library and Tableau software were used to produce graphics.^33^^,^^34^ The data is presented in a narrative way.

### Role of the funding source

All authors had access to the data presented in the study and were involved in the decision to submit for publication. The funders of the study had no role in study design, data collection, data analysis, data interpretation. The funders had a role in writing the report but do not stand to materially benefit from the work.

### Patient public involvement

There was no patient or public involvement due to the ongoing pandemic restrictions.

### Ethical approval

None required.

## Results

**Figure 1:**
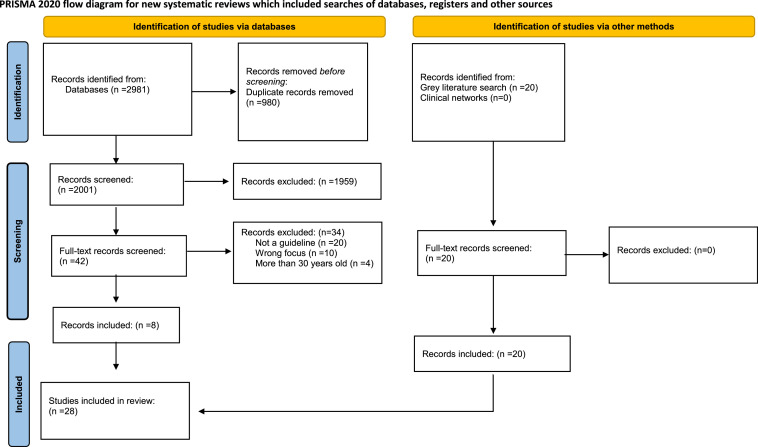
**PRISMA diagram**. This flow diagram depicts the number of records identified included and excluded in our review.

From 2981 records screened, 28 CMGs met the inclusion criteria (Figure 1).^8^^,^^35^, ^36^, ^37^, ^38^, ^39^, ^40^, ^41^, ^42^, ^43^, ^44^, ^45^, ^46^, ^47^, ^48^, ^49^, ^50^, ^51^, ^52^, ^53^, ^54^, ^55^, ^56^, ^57^, ^58^, ^59^, ^60^

### Quality

**Figure 2:**
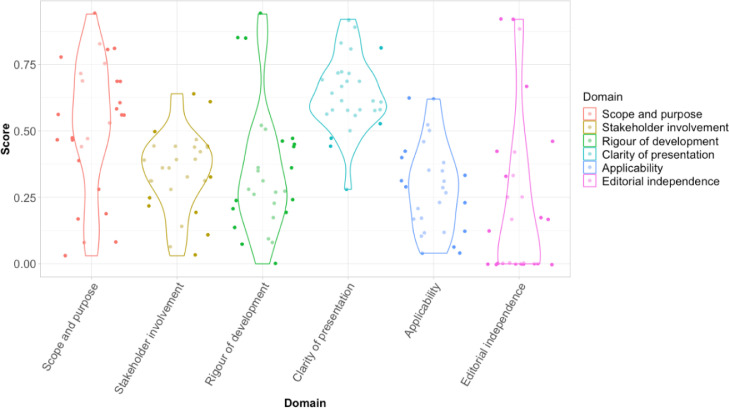
**AGREE II domain scores**. Each violin plot portrays the individual scores of the CMGs in each domain. Each dot represents a CMG proportional score per domain. The width of each curve represents the frequency of CMG scoring in each region. The colours presented correspond to the different domains: Pink- Editorial independence Dark Blue- Applicability Light blue- Clarity of presentation Green- Rigour of development Yellow- Stakeholder involvement Red- Scope and purpose.

The median overall quality of the CMGs was 2 out of 7 points (Table 1). Eighty-six percent (24/28) were of low quality (score ≤ 3), two (7%) of medium (scores 4–5), and two (7%) of high quality (score 6–7). The higher scoring CMGs were produced by Mexico Ministerio De Salud;^55^ UpToDate;^38^ World Health Organisation Southeast Asia^48^ and República Dominicana Ministerio de Salud Pública.^44^ The highest scoring domains were clarity of presentation [median (IQR): 61% (58–72)] and scope and purpose [median (IQR): 56 (43–70)]. The lowest scoring domain was editorial independence [median (IQR): 15 (0–35)]. Similarly, the domains for rigour of development [median (IQR): 28 (21–45)], applicability [median (IQR): 29 (16–40)] and stakeholder involvement [median (IQR): 36 (2–63)] scored low. (Table 1, Figure 2) The CMGs used different methods to formulate their recommendations. Thirty-six per cent (10/28) used expert consensus only, 11% (3/28) used systematic methods only and 14% (4/28) a combination of consensus and systematic methods, moreover, 43% (12/28) consulted other guidelines and 32% (9/28) did not specifically describe the methods used. Expert groups and clinicians were involved in producing the recommendations of 86% (24/28) of CMGs, and only 21% (6/28) of CMGs provided plans for their recommendations to be reviewed and updated, however, none were living guidelines. Further, 82% (23/28) provided some advice and/or tools on how the recommendations could be put into practice.

**Table 1:** **AGREE II scores**. This table presents the results of each CMG by domain and the overall quality.

CMG	Year	Scope and purpose (%)	Stakeholder involvement (%)	Rigour of development (%)	Clarity of presentation (%)	Applicability (%)	Editorial Independence (%)	Overall quality (1–7)
ACCAR ^44^	2018	61.1	36.1	44.8	55.6	20.8	41.7	1
BCDC ^43^	2017	19.4	11.1	13.5	61.1	12.5	16.7	2
BMS ^27^	2015	47.2	27.8	18.8	72.2	27.1	16.7	1
BSR ^49^	2017	44.4	22.2	51	69.4	10.4	45.8	2
BZLMS ^31^	2017	55.6	30.6	28.1	58.3	45.8	0	1
CDC ^34^	2020	8.3	25	7.3	58.3	12.5	0	1
CMS ^51^	2018	69.4	19.4	24	69.4	39.6	0	2
CRMS ^33^	2014	47.2	30.6	8.3	50	50	0	1
DRMSP ^36^	2014	83.3	41.7	35.4	61.1	62.5	0	4
EMS ^46^	2014	58.3	30.6	20.8	66.7	52.1	0	2
ESMS ^41^	2014	77.8	38.9	26	69.4	29.2	12.5	3
GMS ^53^	2015	69.4	50	16.7	58.3	16.7	0	2
IMOH ^42^	2016	16.7	5.6	9.4	58.3	12.5	16.7	1
JIMA ^35^	2020	52.8	38.9	40.6	58.3	8.3	95.8	2
MMS ^47^	2015	94.4	63.9	93.8	91.7	62.5	87.5	7
MS ^32^	2019	8.3	44.4	36.5	58.3	4.2	41.7	1
PAHO ^45^	2011	52.8	47.2	22.9	52.8	31.3	25	1
PHE ^28^	2014	2.8	2.8	0	27.8	4.2	0	1
PMS ^38^	2015	80.6	44.4	36.5	83.3	29.2	0	3
PMSP ^39^	2015	75	44.4	27.1	47.2	41.7	0	2
PRMS ^48^	2014	27.8	13.9	27.1	72.2	31.3	0	2
PUK ^29^	2014	38.9	36.1	46.9	44.4	6.3	66.7	2
RSMBT ^52^	2020	55.6	33.3	22.9	24	72.2	22.9	2
SMOH ^37^	2016	72.2	38.9	31.3	63.9	29.2	0	3
SPILF ^9^	2015	55.6	38.9	45.8	61.1	16.7	33.3	2
UTD ^30^	2020	47.2	44.4	85.4	80.6	35.4	91.7	6
WHO ^50^	2017	69.4	33.3	43.8	55.6	37.5	25	2
WHOSEA ^40^	2008	80.6	61.1	52.1	88.9	22.9	0	4
**Median**	-	**55.6**	**36.1**	**27.6**	**61.1**	**28.1**	**14.6**	**2**
**Range**	-	**(8–94)**	**(2–63)**	**(0–93)**	**(27–91)**	**(4–62)**	**(0–87)**	**(0–7)**

Abbreviations: ACCAR: Pan-American League of Associations for Rheumatology-Central American Caribbean and Andean Rheumatology Association, BCDC: Bangladesh Centre for Disease Control, BMS: Bolivia Ministerio De Salud, BSR: Brazilian Society of Rheumatology, BZLMS: Brasil Ministério da Saúde, CDC: Centers for Disease Control and Prevention, CMS: Chile Ministerio De Salud, CRMS: Costa Rica Ministerio De Salud, DRMSP: República Dominicana Ministerio de Salud Pública, EMS: Ecuador Ministerio De Salud, ESMS: El Salvador Ministerio De Salud, GMS: Guatemala Ministerio De Salud, IMOH: India Ministry of Health, JIMA: Journal of Indian Medical Association. MMS: Mexico Ministerio De Salud, MS: Medscape, PAHO: Pan American Health Organisation, PHE: Public Health England, PMS; Peru Ministerio De Salud, PMSP: Paraguay Ministerio de Salud, PRMS: Puerto Rico Ministerio De Salud, PUK: Patient UK, RSMBT: Revista da Sociedade Brasileira de Medicine Tropical, SMOH: Spain Ministry of Health, SPILF: Société de Pathologie Infectieuse de Langue Française, UTD: UptoDate, WHO: World Health Organisation, WHOSEA: World Health Organisation Southeast Asia, CMG: Clinical management guidelines, AGREE- Appraisal of guidelines for research and evaluation.

### Availability

**Figure 3:**
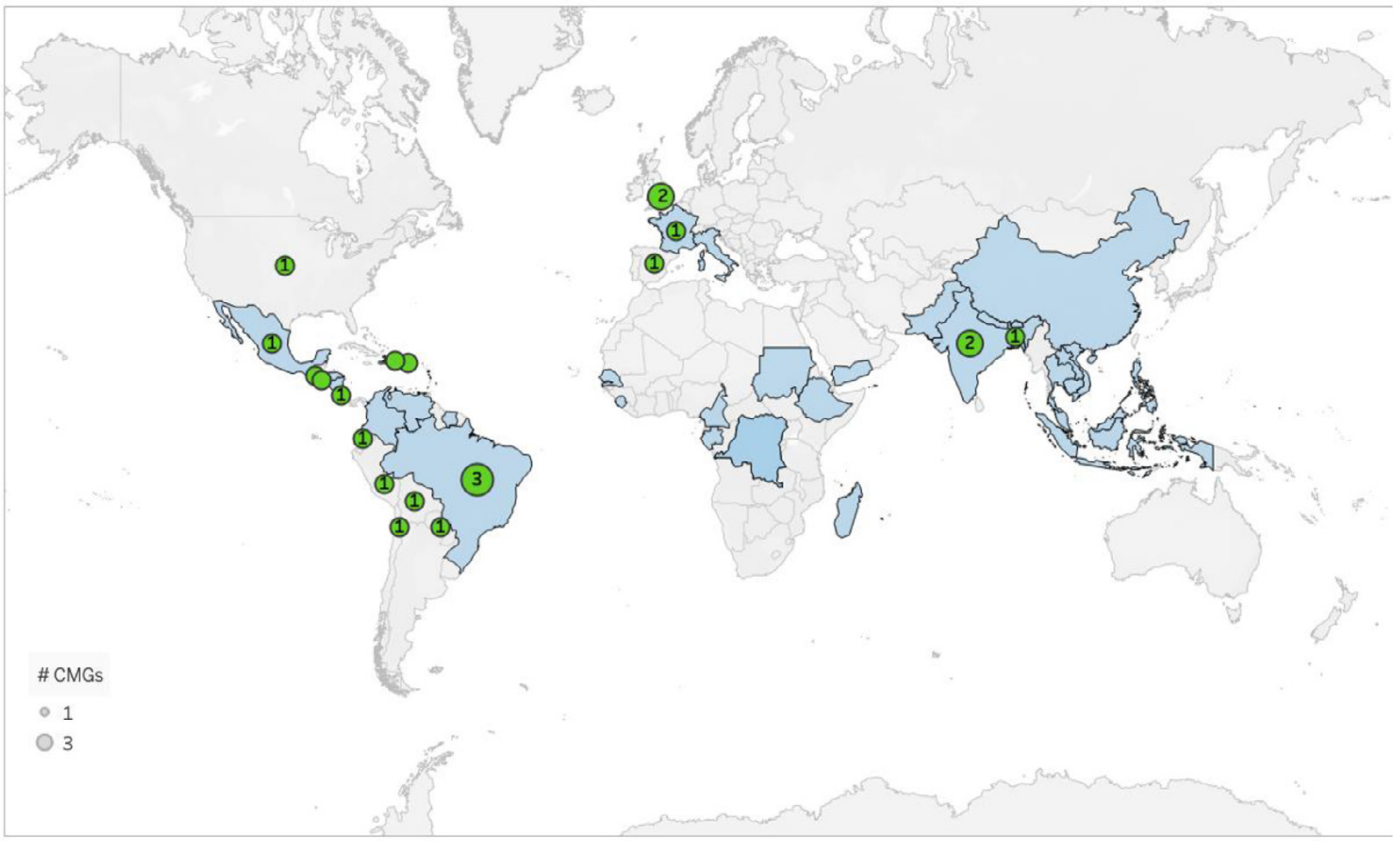
**Chikungunya outbreaks (1999-2020) and geographic distribution of identified CMGs**. The blue shading shows human Chikungunya outbreaks documented as of 1999–2020.^56^ The green dots represent countries with a Chikungunya clinical management guideline (CMGs) and the numbers identified. Additionally, there were three global CMGs produced by the World Health Organisation (WHO), Medscape and Up-to-date, and three regional CMGs produced by the Pan-American Health Organisation (PAHO), WHO South-East Asia (WHOSEA) and Pan-American League of Associations for Rheumatology-Central American Caribbean and Andean Rheumatology Association (ACCAR). (Map adapted from Bettis, A.A and Jackson L.M et al., Plos NTD, 2022^56^).

61% (17/28) of the CMGs were produced in high- or upper-middle income countries.^39^^,^^41^^,^^44^^,^^46^^,^^47^^,^^54^^,^^55^^,^^57^^,^^60^^,^^61^ Fifteen (54%) in Latin America^35^^,^^39^^,^^41^^,^^44^^,^^46^^,^^47^^,^^49^^,^^54^, ^55^, ^56^, ^57^^,^^59^, ^60^, ^61^ four (14%) in Europe,^8^^,^^36^^,^^37^^,^^45^ four (14%) in Asia ^43^^,^^48^^,^^50^^,^^51^ three (11%) in North America^38^^,^^40^^,^^42^ and three for global use. Half of the CMGs were in English (50%, 14/28);^8^^,^^36^, ^37^, ^38^^,^^40^^,^^42^^,^^43^^,^^48^^,^^50^, ^51^, ^52^, ^53^^,^^58^^,^^60^ 43% (12/28) in Spanish^35^^,^^41^^,^^44^, ^45^, ^46^, ^47^^,^^49^^,^^54^, ^55^, ^56^^,^^59^^,^^61^ and 7% (2/28) in Portuguese.^39^^,^^57^ Seventy-five percent (21/28) were produced more than 5 years ago. (Table 1) Further, 82% (23/28) were produced by national organisations, and 18% (5/28) by global/regional organisations.^38^^,^^40^^,^^58^ (Figure 3)

### Inclusivity

Seventy-five percent (21/28) mentioned children, ^8^^,^^35^^,^^37^^,^^38^^,^^43^, ^44^, ^45^, ^46^, ^47^, ^48^, ^49^^,^^51^^,^^52^^,^^54^, ^55^, ^56^, ^57^^,^^59^^,^^61^ 68%, (19/28) pregnant women,^8^^,^^35^^,^^38^^,^^39^^,^^41^^,^^44^, ^45^, ^46^, ^47^, ^48^, ^49^, ^50^, ^51^^,^^53^, ^54^, ^55^^,^^57^^,^^58^^,^^58^^,^^59^ 96% (27/28) people aged >65 years (96%, 27/28),^8^^,^^35^^,^^37^, ^38^, ^39^, ^40^, ^41^, ^42^, ^43^, ^44^, ^45^, ^46^, ^47^, ^48^, ^49^, ^50^, ^51^, ^52^, ^53^, ^54^, ^55^, ^56^, ^57^, ^58^, ^59^, ^60^, ^61^ 21% (6/28) people living with HIV (21%, 6/28)^35^^,^^44^^,^^46^^,^^47^^,^^55^^,^^61^ and 61% (17/28) other comorbidities (61%, 17/28).^8^^,^^35^^,^^37^^,^^38^^,^^40^^,^^42^, ^43^, ^44^^,^^46^^,^^47^^,^^49^^,^^50^^,^^54^, ^55^, ^56^, ^57^^,^^60^ Seventeen (61%) ^36^^,^^37^^,^^39^, ^40^, ^41^, ^42^, ^43^^,^^47^^,^^48^^,^^50^^,^^51^^,^^51^, ^52^, ^53^^,^^59^, ^60^, ^61^^,^^61^^,^^61^ provided some guidance for all groups; and several provided supportive care guidance specifically for pregnant women and children.^8^^,^^29^^,^^35^^,^^38^^,^^41^, ^42^, ^43^^,^^49^, ^50^^,^^52^^,^^53^

### Scope

All provided recommendations for clinical management of acute and chronic CHIKV but with varying level of details (Tables 2 and 3). There was considerable variation amongst CMGs in the recommendations for patients in the acute phase, vulnerable groups and those affected by long-term sequelae. Additionally, there were differences in preventative measures recommended by CMGs to reduce risk of nosocomial and community transmission.

**Table 2:** Summary of acute phase treatment recommendations. **The table presents an overview of the main treatments recommended in the acute phase in each guideline.**

Guidelines	Region	Year	Acute interventions					
			Paracetamol	NSAIDs	Opioids	Antihistamines	Antimalarials	Steroids
ACCAR ^44^	Global	2018	R	RA	RA	NS	NS	R
BCDC ^43^	Asia	2017	R	RA	R	R	NS	RA
BMS ^27^	Latin America	2015	R	R	NS	NS	NS	RA*
BSR ^49^	Latin America	2017	R	RA	R	NS	NS	RA
BZLMS ^31^	Latin America	2017	R	RA	R	NS	NS	R*
CDC ^34^	North America	2020	NS	R	NS	NS	NS	NS
CMS ^51^	Latin America	2018	R	RA	R	R	NS	R
CRMS ^33^	Latin America	2014	R	RA	R	NS	NS	R
DRMSP ^36^	Latin America	2014	R	R	NS	R	NS	RA
EMS ^46^	Latin America	2014	R	R	R	NS	NS	R
ESMS ^41^	Latin America	2014	R	RA	R	NS	NS	RA
GMS ^53^	Latin America	2015	R	R	NS	R	NS	RA
IMOH ^42^	Asia	2016	R	R	R	R	NS	R
JIMA ^35^	Asia	2020	R	R	NS	R	NS	RA
MMS ^47^	Latin America	2015	R	RA	NS	R	NS	RA
MS ^32^	North America	2019	NS	R	NS	NS	NS	RA
PAHO ^45^	Latin America	2011	NS	R	R	NS	NS	R
PHE ^28^	Europe	2014	NS	NS	NS	NS	NS	NS
PMS ^38^	Latin America	2015	R	R	NS	R	NS	RA
PMSP ^39^	Latin America	2015	R	R	R	NS	NS	R
PRMS ^48^	Latin America	2014	R	NS	R	NS	NS	R
PUK ^29^	Europe	2014	R	R	NS	NS	NS	NS
RSMBT ^52^	Latin America	2020	R	RA	R	NS	NS	R*
SMOH ^37^	Europe	2016	R	R*	R	NS	NS	NS
SPILF ^9^	Europe	2015	NS	RA	NR	NS	NS	RA
UTD ^30^	Global	2020	R	R	R	NS	NS	R*
WHO ^50^	Global	2017	R	RA	R	NS	NS	RA
WHOSEA ^40^	Asia	2008	R	R	NS	R	NS	NS
**Total Recommended (R) % (n/n)**			82% (23/28)	54% (15/28)	54% (15/28)	32% (9/28)	0% (0/28)	39% (11/28)

R= recommended, RA= recommended to avoid, NS= not stated.

*For subacute CHIKV.

Abbreviations: NSAID: Non-steroidal Anti-inflammatory Drugs, ACCAR: Pan-American League of Associations for Rheumatology-Central American Caribbean and Andean Rheumatology Association, BCDC: Bangladesh Centre for Disease Control, BMS: Bolivia Ministerio De Salud, BSR: Brazilian Society of Rheumatology, BZLMS: Brasil Ministério da Saúde, CDC: Centers for Disease Control and Prevention, CMS: Chile Ministerio De Salud, CRMS: Costa Rica Ministerio De Salud, DRMSP: República Dominicana Ministerio de Salud Pública, EMS: Ecuador Ministerio De Salud, ESMS: El Salvador Ministerio De Salud, GMS: Guatemala Ministerio De Salud, IMOH: India Ministry of Health, JIMA: Journal of Indian Medical Association. MMS: Mexico Ministerio De Salud, MS: Medscape, PAHO: Pan American Health Organisation, PHE: Public Health England, PMS; Peru Ministerio De Salud, PMSP: Paraguay Ministerio de Salud, PRMS: Puerto Rico Ministerio De Salud, PUK: Patient UK, RSMBT: Revista da Sociedade Brasileira de Medicine Tropical, SMOH: Spain Ministry of Health, SPILF: Société de Pathologie Infectieuse de Langue Française, UTD: UptoDate, WHO: World Health Organisation, WHOSEA: World Health Organisation Southeast Asia.

**Table 3:** Summary of CMG recommendations for treatment of chronic disease symptoms. **The table presents an overview of the main treatments recommended in the chronic phase in each guideline.**

Guidelines	Region	Year	Chronic interventions					
			Analgesia			Steroids	DMARDs	
			Paracetamol	NSAIDs	Opioids		MTX	HCQ
ACCAR ^44^	Global	2018	NS	R	NS	NS	R	R
BCDC ^43^	Asia	2017	NS	NS	NS	NS	NS	R
BMS ^27^	Latin America	2015	NS	R	NS	R	R	NS
BSR ^49^	Latin America	2017	NS	R	R	R	R	R
BZLMS ^31^	Latin America	2017	R	R	R	R	NS	R
CDC ^34^	North America	2018	NS	R	NS	R	NS	NS
CMS ^51^	Latin America	2018	R	R	R	NS	R	NS
CRMS ^33^	Latin America	2014	R	R	NS	R	NS	NS
DRMSP ^36^	Latin America	2014	NS	NS	NS	RA	NS	NS
EMS ^46^	Latin America	2014	NS	R	NS	R	R	NS
ESMS ^41^	Latin America	2014	NS	R	NS	R	R	NS
GMS ^53^	Latin America	2015	NS	NS	NS	RA	NS	NS
IMOH ^42^	Asia	2016	NS	R	NS	NS	NS	R
JIMA ^35^	Asia	2020	NS	NS	NS	R	NS	R
MMS ^47^	Latin America	2015	R	R	NS	RA	NS	NS
MS ^32^	North America	2019	NS	NS	NS	RA	NS	NS
PAHO ^45^	Latin America	2011	NS	R	NS	R	R	RA
PHE ^28^	Europe	2014	NS	NS	NS	NS	NS	NS
PMS ^38^	Latin America	2015	R	NS	NS	NS	NS	NS
PMSP ^39^	Latin America	2015	NS	R	NS	R	R	NS
PRMS ^48^	Latin America	2014	NS	R	NS	NS	NS	NS
PUK ^29^	Europe	2014	R	NS	NS	NS	NS	NS
RSMBT ^52^	Latin America	2020	R	R	R	NS	R	R
SMOH ^37^	Europe	2016	R	NS	NS	R	R	NS
SPILF ^9^	Europe	2015	R	R	R	RA	R	NS
UTD ^30^	Global	2020	R	R	NS	R	R	RA
WHO ^50^	Global	2017	R	R	R	NS	R	NS
WHOSEA ^40^	Asia	2008	R	R	NS	R	NS	NS
Total Recommended (R) % (n/N)			39% (11/28)	64% (18/28)	18% (5/28)	46% (13/28)	65% (11/17)	41% (7/17)
Total Not Recommended (R) % (n/N)			0% (0/28)	0% (0/28)	0% (0/28)	18% (5/28)	0% (0/28)	7% (2/28)

R= recommended, RA= recommended to avoid, NS= not stated.

Abbreviations: NSAID: Non-steroidal Anti-inflammatory Drugs, DMARD: Disease-modifying Antirheumatic Drugs; MTX: Methotrexate; HCQ: Hydroxychloroquine; ACCAR: Pan-American League of Associations for Rheumatology-Central American Caribbean and Andean Rheumatology Association, BCDC: Bangladesh Centre for Disease Control, BMS: Bolivia Ministerio De Salud, BSR: Brazilian Society of Rheumatology, BZLMS: Brasil Ministério da Saúde, CDC: Centers for Disease Control and Prevention, CMS: Chile Ministerio De Salud, CRMS: Costa Rica Ministerio De Salud, DRMSP: República Dominicana Ministerio de Salud Pública, EMS: Ecuador Ministerio De Salud, ESMS: El Salvador Ministerio De Salud, GMS: Guatemala Ministerio De Salud, IMOH: India Ministry of Health, JIMA: Journal of Indian Medical Association. MMS: Mexico Ministerio De Salud, MS: Medscape, PAHO: Pan American Health Organisation, PHE: Public Health England, PMS; Peru Ministerio De Salud, PMSP: Paraguay Ministerio de Salud, PRMS: Puerto Rico Ministerio De Salud, PUK: Patient UK, RSMBT: Revista da Sociedade Brasileira de Medicine Tropical, SMOH: Spain Ministry of Health, SPILF: Société de Pathologie Infectieuse de Langue Française, UTD: UptoDate, WHO: World Health Organisation, WHOSEA: World Health Organisation Southeast Asia.

#### Acute phase of CHIKV infection (Table 2)

Most (96%, 27/28) CMGs recommended symptom-driven clinical management,^35^, ^36^, ^37^, ^38^, ^39^, ^40^, ^41^, ^42^, ^43^, ^44^^,^^46^, ^47^, ^48^, ^49^, ^50^, ^51^, ^52^, ^53^, ^54^, ^55^, ^56^, ^57^, ^58^, ^59^, ^60^, ^61^^,^^61^ with half (50%, 14/28) explicitly stating a lack of effective antivirals.^8^^,^^35^^,^^43^^,^^46^^,^^47^^,^^49^^,^^51^, ^52^, ^53^^,^^57^, ^58^, ^59^, ^60^, ^61^ Nineteen (68%, 19/28)^35^^,^^38^^,^^39^^,^^41^^,^^43^^,^^44^^,^^46^, ^47^, ^48^, ^49^, ^50^, ^51^^,^^53^, ^54^, ^55^, ^56^, ^57^, ^58^, ^59^ provided guidance aimed at different health facility levels depending on disease severity: outpatient care (home based and at the primary care level), secondary level (district hospitals) and at the tertiary level (referral hospitals). The principles of outpatient management were generally consistent amongst the CMGs with recommendations including rest (11/28, 39%),^38^^,^^38^^,^^39^^,^^42^^,^^44^^,^^46^^,^^52^, ^53^, ^54^^,^^58^^,^^59^^,^^61^ hydration (43%, 12/28),^35^^,^^37^, ^38^, ^39^^,^^42^, ^43^, ^44^^,^^47^^,^^52^^,^^58^^,^^59^^,^^61^ cold compresses (11%, 3/28),^44^^,^^51^^,^^57^ antihistamines (39%, 11/28)^38^^,^^44^^,^^46^^,^^48^^,^ ^50^^,^^51^^,^^55^^,^^59^, ^60^, ^61^ and analgesia (96%, 27/28). Fifteen (54%, 15/28)^39^^,^^41^^,^^43^^,^^44^^,^^46^, ^47^, ^48^, ^49^, ^50^, ^51^^,^^53^, ^54^, ^55^^,^^57^^,^^58^ recommended hospitalisation for severe cases; (39%, 11/28)^8^^,^^35^^,^^39^^,^^40^^,^^43^^,^ ^44^^,^^47^^,^^52^^,^^57^^,^^58^^,^^60^ gave guidance regarding managing severe cases. Hospitalisation criteria for severe cases included any signs of haemodynamic instability (46%, 13/28), ^39^^,^^41^^,^^44^^,^^47^, ^48^, ^49^, ^50^, ^51^^,^^53^, ^54^, ^55^^,^^57^^,^^58^ atypical Chikungunya (36%, 10/28),^35^^,^^39^^,^^41^^,^^44^^,^^47^^,^^49^^,^^53^, ^54^, ^55^^,^^58^ severe pain unresponsive to analgesia (25%, 7/28),^41^^,^^43^^,^^44^^,^^47^^,^^48^^,^^50^^,^^51^ signs of haemorrhage (46%, 13/28)^35^^,^^39^^,^^41^^,^^44^^,^^47^, ^48^, ^49^, ^50^, ^51^^,^^53^, ^54^, ^55^ and signs of decompensation from underlying comorbidities (25%, 7/28).^35^^,^^39^^,^^41^^,^^44^^,^^49^^,^^53^^,^^55^ Eighteen CMGs provided a definition for severe cases of Chikungunya.^8^^,^^35^^,^^38^^,^^39^^,^^41^^,^^44^^,^^46^, ^47^, ^48^, ^49^, ^50^, ^51^^,^^53^, ^54^, ^55^^,^^58^^,^^59^^,^^61^ However, only ten clearly stated that this encompassed people experiencing atypical disease manifestations such as respiratory failure, cardiovascular decompensation, myocarditis, acute hepatitis, renal failure, haemorrhage, and/or neurological involvement.^8^^,^^35^^,^^41^^,^^46^^,^^47^^,^^49^^,^^54^^,^^55^^,^^58^^,^^61^ Supportive care recommendations included the use of intravenous fluids (to treat dehydration initially and eventually shock) dehydration and people in shock (55%, 6/11),^35^^,^^43^^,^^51^^,^^51^^,^^53^^,^^54^^,^^58^ haemodynamic monitoring (55%, 6/11),^35^^,^^39^^,^^40^^,^^51^, ^52^, ^53^ blood components (18%, 2/11),^43^^,^^51^ intensive care support as required (9%, 1/11)^8^ and immunoglobulins in CHIKV-related polyneuropathy (4%, 1/28).

##### Antimalarials

None of the CMGs advocated for use of empiric antimalarials for acute infection, but two (10%)^8^^,^^50^ advised including malaria in the differential diagnosis. Antimalarial chloroquine derivatives were discussed for the treatment of long-term chronic manifestations in (24%, 4/17) CMGs.^39^^,^^43^^,^^50^^,^^51^

##### Analgesia

All CMGs recommended analgesia; 75% (21/28)^35^^,^^37^, ^38^, ^39^^,^^41^^,^^43^^,^^46^, ^47^, ^48^, ^49^, ^50^, ^51^, ^52^, ^53^, ^54^^,^^58^, ^59^, ^60^, ^61^^,^^61^ recommended paracetamol as first line treatment for pain and for its antipyretic properties. Four CMGs (14%, 4/28)^38^^,^^39^^,^^48^^,^^54^ advised that paracetamol can cause hepatoxicity. One advised no more than four grams per 24 h,^39^ another to avoid in patients with liver disease,^38^ and one advised monitoring of patients whilst on treatment.^54^ Further, 36% (10/28)^8^^,^^39^^,^^41^^,^^46^^,^^53^^,^^54^^,^^58^, ^59^, ^60^, ^61^ advised escalating to tramadol, codeine or opiates, alone or in combination with paracetamol, for uncontrolled pain. Two (7%, 2/28)^39^^,^^57^ recommended dipyrone for mild pain. There was varying and contradictory advice regarding the use of non-steroidal anti-inflammatory drugs (NSAIDs) in the acute phase. While 54% (15/28) recommended the use of NSAIDs,^35^^,^^37^^,^^38^^,^^40^^,^^42^, ^43^, ^44^, ^45^, ^46^, ^47^, ^48^^,^^50^^,^^53^^,^^54^^,^^61^ 75% (21/28) advised avoiding salicylates in adults during the acute phase due to risk of haemorrhage,^8^^,^^35^^,^^38^, ^39^, ^40^, ^41^^,^^43^, ^44^, ^45^, ^46^, ^47^, ^48^, ^49^, ^50^^,^^53^, ^54^, ^55^, ^56^, ^57^, ^58^, ^59^ and 40% (11/28) advised against NSAIDs.^8^^,^^39^^,^^41^^,^^49^^,^^51^^,^^52^^,^^55^^,^^57^, ^58^, ^59^, ^60^ Two CMGs (7%, 2/28)^30^^,^^50^ did not mention the use of NSAIDs in acute management. One CMG stated a lack of evidence to support the avoidance of NSAIDs.^38^ Eight (29%, 8/28)^8^^,^^35^^,^^41^^,^^47^^,^^52^^,^^54^, ^55^, ^56^ recommended excluding co-infection with dengue prior to NSAID administration. However, the risk of haemorrhage is rare in CHIKV in comparison to dengue and more than half advised the cautious use of NSAIDs (due to the risk of precipitating acute kidney failure) in acute CHIKV infection. NSAIDs have been recognised as a risk factor for severe disease.

##### Corticosteroids

The recommendations for corticosteroids were also heterogenous. Eleven (39%, 11/28)^38^^,^^41^^,^^47^^,^^50^^,^^52^, ^53^, ^54^^,^^56^^,^^59^^,^^60^^,^^69^ advised a short course of corticosteroids if no response to analgesia. A number of additional indications were given, including severe joint pain refractory to analgesia (80%, 8/10);^38^^,^^41^^,^^47^^,^^50^^,^^52^, ^53^, ^54^^,^^59^ highly inflammatory forms (exhibiting bursitis, severe synovitis, joint swelling or persistently raised inflammatory markers) (30%, 3/10);^38^^,^^59^^,^^60^ disabling arthritis/arthralgia (40%, 4/10)^38^^,^^47^^,^^50^^,^^60^ or when NSAIDs are contraindicated (10%, 1/10).^59^ Prednisolone was the most commonly recommended (50%, 5/10),^38^^,^^47^^,^^52^^,^^59^^,^^60^ but with variations in recommend dosing for adults ranging from 10 mg to 20 mg per day (60%, 3/5) based on clinical judgment,^38^^,^^52^^,^^59^ to escalation to 0.5 mg/kg/day (80%, 4/5)^38^^,^^47^^,^^59^^,^^60^ for severe cases. Four CMGs (80%, 4/5)^38^^,^^47^^,^^59^^,^^60^ provided guidance on the duration, ranging from 5 days (60%, 3/5)^38^^,^^47^^,^^59^ to weaning over 10 days to 1–2 months for severe cases (40%, 2/5).^38^^,^^60^ Two CMGs (40%, 2/5) recommended that the duration should not exceed one month.^38^^,^^59^ Although 80%, (4/5)^38^^,^^47^^,^^59^^,^^60^ of CMGs providing corticosteroid guidance, advised on tapering down steroid doses, only one stated the risk of symptom rebound if withdrawn too abruptly.^60^ In contrast 43% (12/28)^8^^,^^35^^,^^40^^,^^43^^,^^44^^,^^46^^,^^49^^,^^51^^,^^55^^,^^57^^,^^58^^,^^61^ of CMGs advised against steroid use in the acute phase of infection. Only a minority gave justifications for avoidance, stating either a lack of evidence (8%, 1/12),^40^ lack of benefit regardless of form of administration (8%, 1/12)^58^ or a risk of rebound symptoms (8%, 1/12).^61^ One CMG advised use of short-term corticosteroids in the acute phase, for individuals with refractory pain, while also advising against the use in the acute phase.^35^

Four CMGs (14%)^35^^,^^38^^,^^39^^,^^60^ recommended to use steroids in the subacute phase to treat symptoms refractory to NSAIDs, moderate pain and arthritis/arthralgia/tenosynovitis. Three of these (75%, 3/4)^38^^,^^39^^,^^60^ advised that prednisolone was first-line, for up to one month. One CMG (25%, 1/4)^39^ provided recommendations on how to assess improvement (ability to walk without assistance; satisfactory pain control) to guide dose and duration.

#### Chronic phase of CHIKV infection

26 (93%)^8^^,^^35^^,^^38^, ^39^, ^40^, ^41^, ^42^, ^43^, ^44^, ^45^, ^46^, ^47^, ^48^, ^49^, ^50^, ^51^, ^52^, ^53^, ^54^, ^55^, ^56^, ^57^, ^58^, ^59^, ^60^, ^61^ provide guidance on the management of long-term sequelae. Recommendations included analgesia, corticosteroids, disease modifying anti-rheumatic drugs (DMARDs) and antimalarial chloroquine derivatives. Five CMGs (18%)^8^^,^^39^^,^^48^^,^^57^^,^^60^ advised using quantitative scoring measures (visual scales, clinical scores, and structured questionnaires) to measure outcomes such as pain, joint involvement, quality of life and functional capacity in adults. The most commonly recommended tool to assess the severity and response to treatment was a visual analogue scale (VAS) (80%, 4/5).^8^^,^^39^^,^^57^^,^^60^ The Routine Assessment of Patient Index Data 3 (RAPID3), Disease Activity Score-28 (DAS28) and Douleur Neuropathique 4 (DN4) were other scales recommended to assess the functional impact of pain and neuropathic pain.^8^^,^^60^

##### Analgesia

Twenty-four CMGs (86%)^8^^,^^35^, ^36^, ^37^, ^38^, ^39^^,^^41^^,^^42^^,^^45^, ^46^, ^47^, ^48^, ^49^, ^50^^,^^52^, ^53^, ^54^, ^55^, ^56^, ^57^, ^58^, ^59^, ^60^ recommended analgesia, primarily NSAIDs (75%, 18/24) ^8^^,^^35^^,^^38^^,^^39^^,^^41^^,^^47^, ^48^, ^49^, ^50^^,^^52^, ^53^, ^54^, ^55^, ^56^^,^^56^, ^57^, ^58^^,^^60^ paracetamol (45%, 11/24)^8^^,^^37^, ^38^, ^39^^,^^41^^,^^45^^,^^46^^,^^48^^,^^55^^,^^58^^,^^60^ and opiates (21%, 5/24) for managing chronic pain.^8^^,^^39^^,^^57^^,^^58^^,^^60^ Only a few (13%, 3/24)^57^^,^^59^^,^^60^ provided guidance on the duration of treatment, ranging from reassessing after four,^57^ eight^60^ to ‘several’ weeks.^59^

##### Corticosteroids

13 (46%) CMGs recommended steroids for the management of chronic phase.^35^^,^^38^^,^^42^^,^^43^^,^^45^^,^^47^, ^48^, ^49^, ^50^^,^^53^^,^^57^^,^^58^ The most common indication was for disabling peripheral arthritis/arthralgia refractory to other treatments (62%, 8/13),^35^^,^^39^^,^^42^^,^^45^^,^^47^^,^^50^^,^^53^^,^^58^ followed by neuropathic symptoms (8%, 1/13),^57^ and those experiencing arthritis/arthralgia, tendinitis, or bursitis with evidence of severe synovitis, joint swelling and persistent elevation of inflammatory markers (8%, 1/13).^38^ Four (31%) recommended prednisolone,^38^^,^^39^^,^^47^^,^^57^ with 75% (3/4) specifying a dosage of 0.5 mg/kg/day.^38^^,^^39^^,^^47^ There was considerable variation in the recommended duration with CMGs advising courses of five,^38^ ten,^47^ 21,^39^ or 28 days.^43^ One CMG advised 5 to 20 mg/day for musculoskeletal and neuropathic symptoms for six to eight weeks, with a weaning period.^57^ Four (31%) CMGs recommended oral steroids,^39^^,^^43^^,^^45^^,^^57^ 15%, (2/13) advised that local intra-articular injections may be beneficial.^45^^,^^53^ In contrast, 18% (5/28) CMGs ^8^^,^^40^^,^^44^^,^^55^^,^^61^ advised against the use of corticosteroids in the chronic phase due to risk of symptom rebound (20%, 1/5),^61^ or lack of published evidence (20%, 1/5).^40^

##### DMARDs

Seventeen (61%) CMGs^8^^,^^35^^,^^38^^,^^39^^,^^43^^,^^45^^,^^47^^,^^49^, ^50^, ^51^, ^52^, ^53^, ^54^^,^^57^, ^58^, ^59^, ^60^ provided guidance on the use of DMARDs to treat long-term sequalae and chronic symptoms with heterogenous recommendations. Eleven of these (65%) recommended methotrexate as first line therapy;^8^^,^^35^^,^^38^^,^^45^^,^^47^^,^^49^^,^^52^, ^53^, ^54^^,^^58^^,^^59^ whereas others (24%, 4/17) recommended chloroquine/ hydroxychloroquine.^39^^,^^50^^,^^50^^,^^51^ One (6%, 1/17) recommended methotrexate for inflammatory joint disease (moderate or severe disease affecting more than five joints) and hydroxychloroquine reserved for less severe forms.^60^ Another CMG noted that there was a lack of data comparing the efficacy of methotrexate and hydroxychloroquine, but recommended hydroxychloroquine as a safer choice due to its anti-inflammatory and possible antiviral effects.^39^ Two CMGs recommended methotrexate either alone or in combination with another DMARD, such as sulfasalazine or chloroquine.^52^^,^^57^ One CMG divided chronic manifestations into post-Chikungunya rheumatoid arthritis/arthralgia (methotrexate first line), post-Chikungunya spondyloarthritis (NSAIDS first line) and post-Chikungunya undifferentiated polyarthritis (NSAIDs first line; corticosteroids second line).^8^ Five CMGs (18%) provided guidance for neuropathic pain management using amitriptyline, pregabalin, gabapentin and carbamazepine.^8^^,^^39^^,^^51^^,^^57^^,^^60^

#### Vulnerable populations

##### Pregnant women

Most CMGs (75%, 21/28) addressed management of CHIKV infection during pregnancy.^8^^,^^35^^,^^37^, ^38^, ^39^^,^^41^^,^^42^^,^^44^, ^45^, ^46^, ^47^, ^48^, ^49^, ^50^, ^51^^,^^53^, ^54^, ^55^^,^^57^, ^58^, ^59^ Yet, only 6/21 (29%) CMGs provided specific guidance on CHIKV symptom control during pregnancy.^8^^,^^44^^,^^47^^,^^49^^,^^58^^,^^59^ Four (67%) recommended paracetamol,^8^^,^^44^^,^^58^^,^^59^ one recommended amoxicillin if febrile (>38.5C) (17%, 1/6)^8^ and 50% (3/6) recommended avoiding NSAIDs and aspirin (50%) citing the risk of ductus arteriosus closure, fetal renal failure and intrauterine death.^8^^,^^58^^,^^59^ Twelve (57%, 12/21) recommended referral to health services for monitoring of mother and child, but the level of monitoring advice varied.^9^^,^^29^^,^^35^^,^^38^^,^^40^, ^41^, ^42^^,^^47^, ^48^, ^49^^,^^51^^,^^52^ One CMG recommended admitting all pregnant women with suspected Chikungunya in the last trimester,^41^ one specified from week 38.^54^ Two CMGs (16%, 2/12)^39^^,^^55^ recommended daily monitoring of all pregnant women with suspected Chikungunya and three (25%, 3/12) recommended obstetric referral if in the final trimester.^8^^,^^58^^,^^59^ Delaying delivery beyond the highly viraemic stage with an aim to prevent mother-to-child transmission (MTCT) was advised in 33% (4/12) CMGs. One (8%),^8^ advised tocolytics, another (8%, 1/12)^41^ postponement of elective caesarean section. Whereas four (19%) CMGs advised that caesarean sections do not prevent mother-to-child transmission.^8^^,^^44^^,^^51^^,^^55^

##### Neonates and children

Many (79%, 22/28) CMGs highlighted that children and neonates are at higher risk of developing severe CHIKV infection and advised referral to hospital, but the referral criteria varied.^8^^,^^35^^,^^37^^,^^39^^,^^41^^,^^43^, ^44^, ^45^, ^46^, ^47^, ^48^, ^49^, ^50^, ^51^, ^52^, ^53^, ^54^, ^55^, ^56^, ^57^, ^58^, ^59^, ^60^ Four (18%)^8^^,^^47^^,^^58^^,^^59^ recommended in-patient monitoring of neonates born to mothers with suspected CHIKV for seven days. The guidance differed for neonates born to mothers with confirmed infection, with three CMGs (14%, 3/22) advising five-days of in-hospital monitoring,^8^^,^^58^^,^^59^ one at least seven days.^47^ One (5%, 1/22) recommended that symptomatic neonates should be cared for in a neonatal intensive care unit.^44^ Four (18%, 4/22) recommended to admit infants (less than 12 months) who were assessed as at risk of CHIKV infection for observation.^35^^,^^43^^,^^46^^,^^51^ Eight CMGs (29%, 8/28) highlighted risk of Reye's syndrome associated with aspirin use in children younger than 12 years old.^41^^,^^47^, ^48^, ^49^^,^^55^^,^^56^^,^^59^ Four CMGs (18%, 4/22) ^8^^,^^35^^,^^58^^,^^59^ advised against NSAIDs in children younger than 3 months old, and three (11%) against codeine use in children younger than 12 years.^8^^,^^58^^,^^59^ One CMG (4%) advised against use of dipyrone in infants younger than three months or weighing less than 5 kg.^60^ Four (18%, 4/22) CMGs advised that there was no risk of transmission through breastmilk.^44^^,^^50^^,^^51^^,^^53^

##### Older adults and those with comorbidities

Most, (96%, 27/28) CMGs^8^^,^^35^^,^^36^^,^^38^^,^^40^, ^41^, ^42^, ^43^, ^44^, ^45^, ^46^, ^47^, ^48^, ^49^, ^50^, ^51^, ^52^, ^53^, ^54^, ^55^, ^56^, ^57^^,^^59^, ^60^, ^61^, ^62^ included some but limited advice for older adults and those with comorbidities. The definition of older adults varied from over 60 to over 65 years of age. While 81% (22/27)^8^^,^^35^^,^^37^^,^^38^^,^^41^, ^42^, ^43^, ^44^, ^45^^,^^47^^,^^48^^,^^50^^,^^52^, ^53^, ^54^, ^55^, ^56^, ^57^, ^58^, ^59^^,^^61^ advised that older adults were at increased risk of severe/atypical disease and death, only seven (26%)^43^^,^^47^^,^^47^^,^^48^^,^^51^^,^^54^^,^^59^ recommended referral to hospital for monitoring. Two (22%, 2/9) advised that those over 60 years old had a 50-times higher mortality risk compared to younger adults.^47^^,^^49^ One CMG highlighted that people over 65 years old were at higher risk of CHIKV complications including dementia, paralysis and kidney disease.^50^ Seventeen (61%) CMGs advised that people with pre-existing chronic conditions (e.g. diabetes, hypertension, heart disease) were at higher risk of severe and atypical CHIKV disease, and deterioration due to decompensation of their pre-existing condition.^8^^,^^35^^,^^37^^,^^38^^,^ ^40^^,^^42^^,^^44^^,^^46^^,^^49^^,^^50^^,^^54^, ^55^, ^56^, ^57^, ^58^^,^^60^ Of these, nine recommended a lower threshold for referral to hospital, and three close monitoring of these high-risk patients. In keeping with general guidance, five CMGs (45%, 5/11) advised prescribing NSAIDs with caution in patients with comorbidities due to risk of renal impairment and bleeding.^39^^,^^44^^,^^46^^,^^54^^,^^60^

#### Prevention of onward transmission

Twenty (71%) CMGs^8^^,^^35^^,^^37^^,^^38^^,^^41^^,^^43^^,^^44^^,^^46^, ^47^, ^48^, ^49^, ^50^, ^51^, ^52^, ^53^, ^54^, ^55^, ^56^, ^57^^,^^59^ provided recommendations regarding the prevention of nosocomial and hospital transmission. Recommendations included use of mosquito repellents (50%, 10/20),^8^^,^^37^^,^^41^^,^^46^^,^^47^^,^^49^^,^^50^^,^^53^^,^^56^^,^^57^ protective clothing (35%, 7/20),^37^^,^^46^^,^^47^^,^^49^^,^^56^^,^^57^ mosquito nets (60%, 12/20),^8^^,^^43^^,^^46^, ^47^, ^48^, ^49^, ^50^^,^^53^^,^^55^, ^56^, ^57^^,^^59^ and isolation (25%, 5/20) of the patient and those in proximity to the patient. It was recommended to continue these measures throughout the febrile illness. In contrast, two CMGs stated that there was no requirement to segregate the infected patient in a household.^50^^,^^51^ Only three CMGs (15%, 3/20) advised on the risk of blood-borne transmission^8^^,^^44^^,^^52^ with one specifying highest risk within the first five days of symptomatic infection.^44^ Two (10%, 2/20) highlighted risk of transmission via organ/tissue transplantation.^44^^,^^52^ Seven CMGs (35%)^8^^,^^35^^,^^50^^,^^51^^,^^54^^,^^56^^,^^57^ recommended vector control measures around the hospital/homes of infected patients, using insecticides,^8^^,^^54^ fumigation^35^ and eradication of breeding sites.^8^^,^^56^ Thirteen (65%) advised notification to public health authorities.^35^^,^^37^^,^^37^^,^^41^^,^^46^, ^47^, ^48^^,^^50^^,^^51^^,^^53^^,^^54^^,^^54^, ^55^, ^56^

## Discussion

This systematic review highlights limited availability of high-quality guidelines for the management of Chikungunya infection globally. In those identified in this review, we found significant heterogeneity in recommendations. Although there was a consensus on the symptomatic treatment of acute non-severe illness, there was a general lack of detailed management advice to guide supportive care. Furthermore, there was significant heterogeneity in recommendations about the use of corticosteroids, with some advocating for their use in the acute phase, while a third advising against during the acute phase. The duration of steroid treatment for both acute and chronic disease was another point of contention between CMGs. There was also variable and contradictory advice on the use of NSAIDs in the acute phase. Further, a lack of standardisation within the classification of the disease stages of sub-acute, acute, and chronic disease may impact on the recommendations and on the management of patients.

The heterogeneity observed, including in the recommendations for the use of corticosteroids in these CMGs reflect the uncertainty for the management of acute Chikungunya and scarcity of research. One prospective randomized parallel group study of 120 patients with acute CHIKV in South India demonstrated that the addition of corticosteroid to NSAIDs reduced pain and improved quality of life.^63^ Another study of 19 cases observed an improvement in mobility with short term corticosteroids in acute CHIKV, however noted that there was a risk of rebound symptoms after treatment cessation.^64^ Several reviews advise caution against the use of corticosteroids due to the risks of rebound symptoms and immunosuppression causing potential disease exacerbation.^65^^,^^66^

Although CHIKV has a low overall mortality risk, it can cause significant morbidity and be fatal for more vulnerable population groups, through associated complications or by triggering decompensation in patients with pre-existing co-morbidities.^67^ Joint pain caused by CHIKV infection may be debilitating, limiting daily activities.^8^ Polyarthralgia is recurrent in 30–40% of infected individuals and may persist for years.^3^^,^^24^^,^ The risk of prolonged sequelae in populations in lower resourced settings can have a profound impact on livelihoods, with wider socio-economic impact on individuals, their families and society. Public health interventions adopted during the COVID-19 pandemic may have had a negative impact on vector surveillance and control.^68^^,^^69^ As we are transitioning out of the pandemic, we need to prepare to shift resources to identify and mitigate the wider pandemic consequences and strengthen our capacity to respond to future epidemics. Considering the high number of people affected by and at risk of CHIKV infection, the scarcity of treatments, and heterogeneous and at times contradictory supportive care recommendations identified are reasons for concern.

While many CMGs identified children and neonates as high-risk groups for more severe illness, referral and monitoring criteria differed, and the advice on how to reduce risk of MTCT during delivery was limited. Although there are novel approaches suggested to prevent the risk of MTCT, such as anti-CHIKV hyperimmunoglobulins, the evidence-base is limited and there is currently no approved treatment.^70^, ^71^, ^72^ The CMGs were also limited in specific advice for older people and for those with co-morbidities, both of whom are at higher risk of more severe disease.^2^

Although most CMGs provided recommendations for post-acute follow-up care and treatment of chronic complications, the recommendations were heterogenous and with limited evidence provided to support them. There was variation in the recommendations on use of DMARDs, especially for hydroxychloroquine and methotrexate in the management of chronic Chikungunya. A study examined combination DMARD therapy versus hydroxychloroquine treatment in 72 patients with post-Chikungunya arthritis and found that a combination of DMARDs were superior to hydroxychloroquine monotherapy with improvements in disability, reduction in pain and disease activity.^73^ Despite acknowledging this lack of benefit, four CMGs recommended hydroxychloroquine as a first line DMARD.^39^^,^^43^^,^^50^^,^^51^ Existing interventional clinical studies are limited, and with a lack of standardised methodologies, the ability to conduct meta-analyses is restricted, thus limiting our evidence base in determining the most effective therapies for treating chronic manifestations of CHIKV infection.^74^

This review is not without limitations. Despite a systematic search, additional local guidelines may exist. Approximately half of the included CMGs were in a language other than English, and although these were assessed by a reviewer with good knowledge of that language, there may have been slight nuances lost in translation. Furthermore, the AGREE-II tool^32^ assess methodological aspects relevant to guideline development, but not the validity of the clinical management recommendations, conclusions about the validity of the clinical guidance made can therefore not be derived from the AGREE assessment.^32^

Despite these limitations, this review identifies concerning gaps and disparities within the CMGs. Firstly, there is an issue of accessibility, with the two highest quality CMGs identified in this review not being freely accessible^38^^,^^75^ Developing CMGs is resource intensive, and given the changing epidemiology, it requires systems for regular reviews, updates, and re-dissemination. CHIKV disproportionally impacts lower resourced settings, where such resources may not be readily available. Further, other infections may take priority when there is international pressure and/or funding to develop research and guidelines (e.g., SARS-CoV-2, HIV, malaria). International high-quality and easily accessible CMGs, that can be adapted to different settings may fill this gap, as long as implementation is supported in different resourced settings. A living guideline framework, such as the platform developed by WHO during the COVID-19 pandemic providing a living covid-19 CMG, regularly updated by a wider range of expert stakeholders, may improve availability of up-to-date guidelines.^76^ WHO is a normative body and their guidelines are adopted by many healthcare systems globally,^77^ besides saving local resources, it also facilitates standardisation of care between sites. Wide stakeholder engagement, including clinicians from endemic regions and patient groups is important and may improve inclusivity and applicability, and ensure that guidelines address local needs. Further research to explore implementation and impact of CMGs in different setting is recommended to inform CMG development frameworks.

Our findings highlight a lack of high-quality, standardised Chikungunya CMGs globally, especially for those at higher risk of severe illness. Given the risk that CHIKV infection poses globally and in particular to vulnerable groups such as children, pregnant women, older adults and those with co-morbidities, it is essential that existing guidelines are updated with latest evidence and inclusive of all risk groups. Our data also highlights an urgent need for trials to identify optimal treatment and supportive care strategies for different population groups to improve long term outcomes and for new evidence to be incorporated into guidelines. A new ‘living guideline’ framework for infectious diseases is recommended,^78^ to improve availability of up-to-date guidelines, developed using robust methodologies, co-developed by diverse stakeholders to support inclusivity and implementation to improve long term patient outcomes in any resourced settings.

## Contributors

AD, VC, LS, SL, EH, STJ, EW, MM, IR developed the study protocol. EH, AD carried out the database search with input from MM, EW and IR. EW, MM, IR, AD, EC conducted the grey literature search.

EW, RJ, AD, MM screened articles for inclusion. AD, EW, IR, RJ, MM extracted the data and completed the risk of bias analysis. EW, MM, RJ, DD, SL, and IR led on data analysis, and presentation of the results. EW, MM, and IR verified the underlying data. All co-authors informed the interpretation of the findings. EW led on writing the manuscript with inputs from LS, IR, MM, DD, RN, PPJ, ES, KG, and MC. LS, PWH, TF and STJ provided overall supervision, leadership, and advice. PWH, HG, STJ, TF, PWH, LS, AD, and LB conceptualised the project. All authors had full access to the data presented in the study, reviewed, approved the final version of the manuscript and accept responsibility to submit for publication.

## Data sharing statement

All data generated or analysed during this study that are not available in this manuscript, or the supplementary file can be reasonably requested from the corresponding author.

## Declaration of interests

All authors have completed the ICMJE uniform disclosure form. Peter Hart is a senior research advisor and Helen Groves is a research manager at the Wellcome Trust, which provided part of the funding for this work, but, neither had a role in data collection, analysis nor interpretation of the findings. Wellcome supports a range of research funding activities including awards made to ISARIC.

## Appendix Supplementary materials

Image, application 1
