# Supplementary material for: An evaluation of global Chikungunya clinical management guidelines: A systematic review
Source: eClinicalMedicine. 2022 Sep 28;54:101672. doi: 10.1016/j.eclinm.2022.101672 (PMC9526181; doi:10.1016/j.eclinm.2022.101672)
Supplement: Supplementary file 1 [file mmc1.pdf]

Supplementary material: An evaluation of global Chikungunya clinical management guidelines: a systematic review

S1: Search strategy

Updated on 14/10/2021 by an Outreach Librarian at the Bodleian Health Care Libraries, University of Oxford. This review is part of a wider project evaluating the availability, quality, and inclusivity of clinical management guidelines for the management of high consequence infectious diseases (PROSPERO CRD42020167361).

Database: Medline (Ovid MEDLINE® Epub Ahead of Print, In-Process & Other Non-Indexed Citations, Ovid MEDLINE® Daily and Ovid MEDLINE®) 1946 to present

Search Strategy:

----

1 exp clinical pathway/ (7266)

2 exp clinical protocol/ (178784)

3 exp consensus/ (16567)

4 exp consensus development conference/ (12448)

5 exp consensus development conferences as topic/ (2968)

6 critical pathways/ (7266)

7 exp guideline/ (36281)

8 guidelines as topic/ (41609)

9 exp practice guideline/ (29146)

10 practice guidelines as topic/ (125684)

11 (guideline or practice guideline or consensus development conference or consensus development conference, NIH).pt. (46006)

12 (standards or guideline or guidelines).ti,kf,kw. (121468)

13 ((practice or treatment* or clinical) adj guideline*).ab. (45501)

14 (position statement* or policy statement* or practice parameter* or best practice*).ti,ab,kf,kw. (38873)

15 (CPG or CPGs).ti. (6054)

16 consensus*.ti,kf,kw. (29948)

17 ((critical or clinical or practice) adj2 (path or paths or pathway or pathways or protocol*)).ti,ab,kf,kw. (23047)

18 recommendat*.ti,kf,kw. (46828)

19 (care adj2 (standard or path or paths or pathway or pathways or map or maps or plan or plans)).ti,ab,kf,kw. (69406)

20 (algorithm* adj2 (screening or examination or test or tested or testing or assessment* or diagnosis or diagnoses or diagnosed or diagnosing or pharmacotherap* or therap* or treatment* or intervention*)).ti,ab,kf,kw. (19893)

21 1 or 2 or 3 or 4 or 5 or 6 or 7 or 8 or 9 or 10 or 11 or 12 or 13 or 14 or 15 or 16 or 17 or 18 or 19 or 20 (659924)

22 exp Henipavirus/ (809)

23 Henipavirus Infections/ (567)

24 (nipah or hendra).tw. (1353)

25 Monkeypox virus/ or Monkeypox/ (496)

26 (monkeypox or "monkey pox").tw. (803)

27 Chikungunya virus/ or Chikungunya Fever/ (3815)

28 Chikungunya.tw. (5982)

29 "Severe Fever with Thrombocyto* Syndrome".tw. (700)

30 SFTS.tw. (1052)

31 Plague/ (5399)

32 plague.tw. (9601)

33 "black death".tw. (284)

34 "pathogen x".tw. (51)

35 22 or 23 or 24 or 25 or 26 or 27 or 28 or 29 or 30 or 31 or 32 or 33 or 34 (20833)

36 21 and 35 (186)

37 limit 36 to yr="2020 - 2021" (30)

Database: Embase 1974 to present

Search Strategy:

----

1 exp clinical pathway/ (8958)

2 exp clinical protocol/ (107994)

3 exp consensus/ (81891)

4 exp consensus development conference/ (24950)

5 exp consensus development conferences as topic/ (24950)

6 critical pathways/ (8958)

7 exp practice guideline/ (615078)

8 guidelines as topic/ (433775)

9 exp practice guideline/ (615078)

10 practice guidelines as topic/ (368202)

11 (guideline or practice guideline or consensus development conference or consensus development conference, NIH).pt. (0)

12 (standards or guideline or guidelines).ti,kw. (149823)

13 ((practice or treatment* or clinical) adj guideline*).ab. (69128)

14 (position statement* or policy statement* or practice parameter* or best practice*).ti,ab,kw. (55950)

15 (CPG or CPGs).ti. (7254)

16 consensus*.ti,kw. (36376)

17 ((critical or clinical or practice) adj2 (path or paths or pathway or pathways or protocol*)).ti,ab,kw. (34345)

18 recommendat*.ti,kw. (56910)

19 (care adj2 (standard or path or paths or pathway or pathways or map or maps or plan or plans)).ti,ab,kw. (121600)

20 (algorithm* adj2 (screening or examination or test or tested or testing or assessment* or diagnosis or diagnoses or diagnosed or diagnosing or pharmacotherap* or therap* or treatment* or intervention*)).ti,ab,kw. (28786)

21 1 or 2 or 3 or 4 or 5 or 6 or 7 or 8 or 9 or 10 or 11 or 12 or 13 or 14 or 15 or 16 or 17 or 18 or 19 or 20 (1018905)

22 exp henipavirus/ (1485)

23 exp Henipavirus infection/ (608)

24 (nipah or hendra).tw. (1533)

25 monkeypox/ or monkeypox virus/ (861)

26 (monkeypox or "monkey pox").tw. (896)

27 Chikungunya virus/ or chikungunya/ (6073)

28 Chikungunya.tw. (7360)

29 "Severe Fever with Thrombocyto* Syndrome".tw. (758)

30 SFTS.tw. (1258)

31 plague/ (6332)

32 plague.tw. (8453)

33 "black death".tw. (287)

34 "pathogen x".tw. (43)

35 22 or 23 or 24 or 25 or 26 or 27 or 28 or 29 or 30 or 31 or 32 or 33 or 34 (24000)

36 21 and 35 (453)

37 36 (453)

38 limit 37 to yr="2020 - 2021" (83)

Database: Global Health <1973 to 2021 Week 39>

Search Strategy:

----

1 exp consensus/ (140)

2 guidelines/ (55586)

3 (standards or guideline or guidelines).ti. (13730)

4 ((practice or treatment* or clinical) adj guideline*).ab. (6597)

5 (position statement* or policy statement* or practice parameter* or best practice*).ti,ab. (5865)

6 (CPG or CPGs).ti. (532)

7 consensus*.ti. (2284)

8 ((critical or clinical or practice) adj2 (path or paths or pathway or pathways or protocol*)).ti,ab. (1890)

9 recommendat*.ti. (7489)

10 (care adj2 (standard or path or paths or pathway or pathways or map or maps or plan or plans)).ti,ab. (6793)

11 (algorithm* adj2 (screening or examination or test or tested or testing or assessment* or diagnosis or diagnoses or diagnosed or diagnosing or pharmacotherap* or therap* or treatment* or intervention*)).ti,ab. (2152)

12 1 or 2 or 3 or 4 or 5 or 6 or 7 or 8 or 9 or 10 or 11 (82545)

13 exp henipavirus/ (1143)

14 (nipah or hendra).tw. (1155)

15 exp monkeypox virus/ (388)

16 (monkeypox or "monkey pox").tw. (489)

17 exp chikungunya virus/ (4437)

18 Chikungunya.tw. (5218)

19 "Severe Fever with Thrombocyto* Syndrome".tw. (564)

20 SFTS.tw. (524)

21 plague/ (3334)

22 plague.tw. (7648)

23 "black death".tw. (109)

24 "pathogen x".tw. (4)

25 13 or 14 or 15 or 16 or 17 or 18 or 19 or 20 or 21 or 22 or 23 or 24 (15014)

26 12 and 25 (185)

27 26 (185)

28 limit 27 to yr="2020 - 2021" (26)

Scopus

Cluster 3

((TITLE-ABS-KEY ("clinical pathway*" OR "clinical protocol*" OR consensus OR guideline* OR "position statement*" OR "policy statement*" OR "practice parameter*" OR "best practice*") OR TITLE-ABS-KEY (care W/2 (standard OR path OR paths OR pathway OR pathways OR map OR maps OR plan OR plans)) OR TITLE-ABS-KEY (algorithm* W/2 (screening OR examination OR test OR tested OR testing OR assessment* OR diagnosis OR diagnoses OR diagnosed OR diagnosing)) OR TITLE-ABS-KEY (algorithm* W/2 (pharmacotherap* OR therap* OR treatment* OR intervention*)) OR TITLE (standards OR recommendat*))) AND (TITLE-ABS-KEY (henipavirus OR nipah OR hendra OR monkeypox OR "monkey pox" OR chikungunya OR "Severe Fever with Thrombocyto* Syndrome" OR sfts OR plague OR "black death" OR "pathogen x")) AND (LIMIT-TO (PUBYEAR , 2021) OR LIMIT-TO (PUBYEAR , 2020))

Web of Science Core Collection

#1 TOPIC: ("clinical pathway*" OR "clinical protocol*" OR consensus OR guideline* OR "position statement*" OR "policy statement*" OR "practice parameter*" OR "best practice*" OR CPG OR CPGs) OR TOPIC: ((care near/2 (standard or path or paths or pathway or pathways or map or maps or plan or plans))) OR TOPIC: ((algorithm* near/2 (screening or examination or test or tested or testing or assessment* or diagnosis or diagnoses or diagnosed or diagnosing))) OR TOPIC: ((algorithm* near/2 (pharmacotherap* or therap* or treatment* or intervention*))) OR TITLE: (standards OR recommendat*)

#2 TOPIC: (Henipavirus OR nipah or hendra or monkeypox or "monkey pox" OR Chikungunya OR "Severe Fever with Thrombocyto* Syndrome" OR SFTS OR plague OR "black death" OR "pathogen x")

#3 #2 AND #1

#4 **#1 and #2** and **2020** or **2021** (Publication Years)

The WHO Global Index Medicus Regional Libraries https://pesquisa.bvsalud.org/gim/?lang=en

Cluster 3

(tw:("clinical path*" OR "clinical protocol*" or "critical path*" OR "critical protocol*" OR "practice path*" OR "practice protocol*" OR consensus OR guideline* OR standards OR "position statement*" OR "policy statement*" OR "practice parameter*" OR "best practice*" OR CPG OR CPGs OR "care standard" OR "care path*" OR "care map*" OR "care plan*" OR algorithm*)) AND (tw:(Henipavirus OR nipah or hendra or monkeypox or "monkey pox" OR Chikungunya OR "Severe Fever with Thrombocyto* Syndrome" OR SFTS OR plague OR "black death" OR "pathogen x"))

Limits: 2020-2021

S1: Database search results

	Search results (February 2020)	Updated Search results 14/10/2021 (2020-2021 only)
**Database**	**Cluster 3 results**	
Ovid Medline	157	30
Ovid Embase	382	83
Ovid Global Health	157	26
Scopus	725	131
Web of Science Core Collection	722	176
WHO Global Index Medicus	85	12
TOTAL	2228	458
Total after deduplication	1428	278

Google Scholar – Screen the first 10 pages of results

Sorted by relevance:

Cluster 3

(guideline|consensus|standards|"clinical path*”|"clinical protocol*"|"practice path*"|" policy statement*"|"best practice*")(henipavirus|nipah|hendra|monkeypox|chickungunya|"severe fever with thrombocyte* syndrome"|sfts|"black death"|plague|"pathogen x")

https://scholar.google.co.uk/scholar?hl=en&as_sdt=0%2C5&q=%28guideline%7Cconsensus%7Cstandards%7C%22clinical+path*%E2%80%9D%7C%22clinical+protocol*%22%7C%22practice+path*%22%7C%22+policy+statement*%22%7C%22best+practice*%22%29%28henipavirus%7Cnipah%7Chendra%7Cmonkeypox%7Cchickungunya%7C%22severe+fever+with+thrombocyte*+syndrome%22%7Csfts%7C%22black+death%22%7Cplague%7C%22pathogen+x%22%29&btnG=

S1.2: Grey literature search strategy

The search terms below were used to search for chikungunya clinical management guidelines on the ministry of health, public health, and other relevant websites. The first 50 results were examined. If a relevant guideline was found using the first search term, the other search terms were not used.

Search Terms
Clinical management guidelines for chikungunya
Chikungunya clinical management guidelines
Chikungunya clinical guidance
Chikungunya guidelines
Chikungunya guidance
Strategies for management of chikungunya
Clinical practice guidelines for management of chikungunya
Clinical management of chikungunya
Chikungunya clinical management

S1.2.1: Websites searched:

Organisation
IDSA
ERS
ESCMID
BTS

NICE
WHO
US CDC
Africa CDC
Nigeria CDC
China CDC
ECDC
Medscape
Uptodate guidelines
BMJ Best Practice
Taiwan CDC
WHO regional offices

S1.2.2: MoH and national health countries:

Ministry of health countries					
**Latin America**	**Asia**	**Europe**	**Africa**	**Middle East**	**North Americas**
Argentina<br>Brazil<br>Mexico<br>Jamaica<br>Cuba<br>and other countries in the region	Australia<br>Japan<br>South Korea<br>China Taiwan<br>China<br>Malaysia<br>Thailand<br>Iran<br>India<br>Indonesia<br>Nepal	France<br>Italy<br>Germany<br>Russia<br>Turkey<br>Spain	Ghana<br>Nigeria<br>Cameroon<br>South Africa	Saudia Arabia<br>UAE<br>Egypt<br>Jordan<br>Lebanon<br>Tunisia	Canada

	Bangladesh<br>Pakistan<br>Nepal				

S2: Data extraction

S2.1 Data extraction form

Assessment group	Questions
**Availability**	Guideline Title<br>Authors<br>Issuing Organisation<br>Date of latest revision<br>Is the guideline adapted from another source?<br>Publication type (e.g., website, journal article, etc)<br>Intended audience?<br>Geographical aim (e.g., China, UK, USA)<br>What is the setting? (e.g., Low resource setting)
**Inclusivity**	What group of patients is the guideline aimed at?<br>Special populations included?<br>How is Chikungunya to be diagnosed?<br>What settings (hospital, GP)?<br>Any specific exclusions?

	Is there specific guidance for pregnant patients?<br><br>Is there specific guidance for people living with HIV?<br><br>Is there specific guidance for children?<br><br>Is there specific guidance for older people?<br><br>Is there specific guidance for people with comorbidities?<br><br>Are all the above recommendations tied to clear evidence?<br><br>Any other details on population?
**Methodology**	How was the guideline produced?<br>Which key stakeholder groups involved?<br><br>How was the guideline developed?<br><br>How evidence based was each section?<br><br>Are there plans for the guideline to be reviewed?
**Therapeutic and Supportive care recommendations**	Does the guideline recommend a specific therapeutic intervention?<br><br>In treatment or prophylaxis?<br><br>Are there recommendations for how IV access should be obtained?<br>Is there clear advice on the use of vasopressors or inotropes?<br>Is there clear advice on symptom management? e.g., analgesia<br>What analgesia is recommended? Doses? Are NSAIDs recommended?<br><br>What length of analgesia?<br><br>Are steroids recommended? What guidance is there?

Are DMARDS recommended? At what stage?

Indications for rheumatology referral
Is there clear guidance on the role of hemodialysis?
Is supplemental oxygen recommended?
Does the guideline recommend a specific therapeutic intervention? In treatment or prophylaxis?

Are empirical antibiotics recommended?

Are anti-malarials recommended?
Was there a criterion for hospital admission?

Any specific therapeutics to avoid?

Hospitalization criteria for atypical /severe case given?

What criteria given?

chronic phase analgesia time frame given?

What advice given? NSAID and comorbidity
What was time frame for chronic analgesia?

What was pain scale?

Prevention: did they advise informing Public Health?

Any advice for severe disease given?

Severe disease advice?

Notes/other recommendations

Are all the above recommendations tied to clear evidence?
Other comments
